# Experimental assessment of using phase change materials in vapor compression refrigeration systems for condenser pre-cooling

**DOI:** 10.1016/j.heliyon.2024.e40259

**Published:** 2024-11-08

**Authors:** Alireza Riahi, Mohammad Behshad Shafii

**Affiliations:** a Department of Mechanical Engineering, Sharif University of Technology, Tehran, Iran; b Sharif Energy, Water and Environment Institute (SEWEI), Tehran, Iran

**Keywords:** PCM, Refrigeration system, Peak load shaving

## Abstract

The primary objective of this investigation is to empirically assess a new vapor compression cycle while employing phase change material (PCM) energy storage. During off-peak periods, the PCM undergoes charging, and during on-peak hours, it is discharged to cool the refrigerant entering the condenser, thereby enhancing the condenser's overall performance. In contrast to previous studies that exclusively examined the charging or discharging processes of air conditioning (AC) units, this research delves into the entire 24-h charge and discharge cycle. The proposed system is subjected to testing under identical conditions, both without PCM (conventional mode) and with a PCM storage tank, throughout a 24-h period. The PCM storage tank, which utilizes water as its phase change medium, possesses a volume of approximately 300 L and starts at an initial temperature of 25 °C. The outcomes of the study demonstrate notable improvements when incorporating the PCM storage tank. Specifically, the daily coefficient of performance (COP) increases by approximately 7 %, rising from 2.17 to 2.33. Additionally, it leads to a reduction in both the daily available cooling load and daily compressor energy consumption, with decreases of approximately 3.7 % (from 58.4 to 56.2 kWh) and 10.3 % (from 26.9 to 24.1 kWh), respectively.

## 1 Introduction

The increase in the global population and the enhancement of living conditions have played a major role in the surge of energy usage worldwide [1,2]. Of this energy consumption, approximately 40 % is attributed to buildings, with a significant portion allocated to air conditioning and maintaining thermal comfort [3,4]. In 2019, the International Energy Agency (IEA) reported that air conditioning systems dedicated to thermal comfort accounted for around 8.5 % of the total electrical energy consumption. This surge in demand notably affects power plants, particularly during peak hours and warm seasons. During peak hours, residential buildings alone contribute to approximately 15 % of the global electricity consumption for cooling purposes, a figure that can surge to 50 % during hot days, leading to power shortages and increased transmission costs [5,6].

A practical approach to addressing this issue is by shifting electricity usage from peak hours to periods of lower demand [7,8]. This can be accomplished by using thermal energy storage systems, which store cooling energy during off-peak times for later use during peak demand hours [9]. Thermal energy storage systems generally fall into three categories: sensible, latent, and thermochemical [10]. Sensible storage systems store energy by elevating the temperature of the thermal storage medium [11]. Latent energy storage units maintain thermal energy at a constant temperature through the use of phase change materials, resulting in a smaller storage volume requirement for the same thermal energy capacity [12]. Thermochemical energy storage systems offer higher energy density but are less common in building applications due to their complexity and safety concerns; as such, latent energy storage systems are more widely adopted [13,14].

Yamaha and Misaki [15] conducted a study on load shifting in a Japanese office building by incorporating a phase change material (PCM) tank. The charging period was set from 5:00 to 8:00, and the discharging period from 13:00 to 16:00. Their results indicated that 400 kg of PCM, with a melting point of 19 °C, could adequately fulfill the cooling needs of a 73.8 m^2^ building during a hot summer day. Bozanjani and Farid [16] conducted experimental evaluations of a PCM tank's performance in a building during both summer and winter. Their results demonstrated that the use of a PCM tank for cooling energy storage led to energy consumption reductions of up to 30 % in March and April and up to 10 % in January, considering New Zealand's climate conditions. Additionally, they compared the effectiveness of passive (in the walls) and active (thermal storage) modes, with the active mode outperforming the passive mode by reducing energy consumption and electricity costs by approximately 22 % and 32 %, respectively [17]. Comodi et al. [18] investigated an office building's performance utilizing a sensible storage tank based on Singapore's electricity tariff. Their analysis indicated that using storage tanks with capacities between 482 and 2214 m³ across various scenarios led to payback periods ranging from 8.9 to 16 years. Ruddell et al. [19] showed that utilizing thermal energy storage for cooling could lower daily electricity costs by as much as $2.47 in the U.S. conditions. It also significantly decreased electric consumption related to cooling applications by up to 23 % and reduced total electric consumption by up to 15 % during peak hours. In a study by Erdemir and Dincer [20] the impact of peak load shifting, using thermal energy storage, was assessed based on Canadian weather and economic conditions. Their research indicated that using thermal energy storage could cut electricity usage by as much as 45 % during peak periods, leading to a 20 % decrease in cooling expenses. Riahi et al. [21] increasing the COP of the air conditioning system up to 70 % during on-peak hours by adding PCM storage tank to the desired system.

Another approach to reducing the electrical energy consumption of cooling systems is through condenser cooling. By cooling the refrigerant stream entering the condenser, the condenser's operating temperature and, consequently, its operating pressure can be lowered. This leads to a reduction in compressor power and, consequently, energy consumption [22]. In this regard, Wang et al. [23] explored the effects of phase change materials (PCMs) in various locations within a cooling system. They found that cooling the refrigerant stream leaving the condenser to reduce sub-cooling resulted in energy savings of up to 8 %. Additionally, cooling the refrigerant stream entering the condenser improved the coefficient of performance (COP) by up to 6 %. Moreover, cooling the refrigerant stream leaving the evaporator to reduce the degree of superheat increased the COP by up to 7 % [24]. Waly et al. [25] demonstrated that cooling the inlet air to the condenser for an air conditioning unit improved the COP from 36 % to 59 % using various cooling methods under Kuwait's weather conditions. In this regard, Ibrahim et al. [26] reported that reducing the temperature of the condenser inlet air stream by 4° enhanced the COP by approximately 21.4 %. Riahi et al. [27] evaluated the impact of cooling the condenser inlet refrigerant using four different PCMs, based on Tehran's weather conditions. Their study revealed that the use of SP224A as a PCM with a 150-L storage tank reduced peak load demand by up to 76.3 %. In another study [28], the effect of various mechanical parameters of PCM storage tanks on the performance of air conditioning systems was evaluated. The study reported that the use of SP224A as a PCM reduced compressor power consumption by approximately 23.38 %.

The majority of studies in the existing literature have focused on improving the performance of vapor compression cycles by altering components like the compressor, condenser, expansion valve, and selecting different refrigerants [29]. Few studies have investigated the use of Phase Change Materials (PCMs) in refrigeration cycles to enhance performance and reduce peak load demand. Additionally, some investigations have examined the impact of integrating PCMs at various points within the vapor compression cycle. However, these studies have predominantly focused on the charging phase, with minimal attention given to the discharging process. Furthermore, there has been a notable absence of research regarding the influential factors and dynamic assessment of such systems. In this study, an experimental assessment of a novel vapor compression cycle is carried out, incorporating a PCM storage tank. The PCM serves as a means of storing cooling energy during off-peak periods, subsequently enhancing the condenser's performance by utilizing the stored thermal energy during on-peak hours. Consequently, the overall efficiency of the vapor compression cycle experiences enhancements. In the proposed system, the PCM is charged (frozen) during off-peak hours and discharged (melted) during peak hours to cool the refrigerant before it enters the condenser. Unlike earlier studies that mainly concentrated on either the charging or discharging phases of PCM storage in air conditioning units, this research provides a thorough experimental evaluation of an AC unit that incorporates a PCM storage tank, covering both the complete charging and discharging processes over a 24-h period.

## 2 System description

The conventional vapor compression cycle consists of four main components: an evaporator, a condenser, a compressor, and a throttle valve. The compressor raises the pressure and temperature of the refrigerant stream, which then flows into the condenser, where it dissipates heat to the surrounding environment. Subsequently, the refrigerant stream passes through the throttle valve, causing a reduction in both pressure and temperature. This results in the refrigerant entering the evaporator in a two-phase state, producing cooling energy. The refrigerant then returns to the compressor to complete the cycle.

**Fig. 1:**
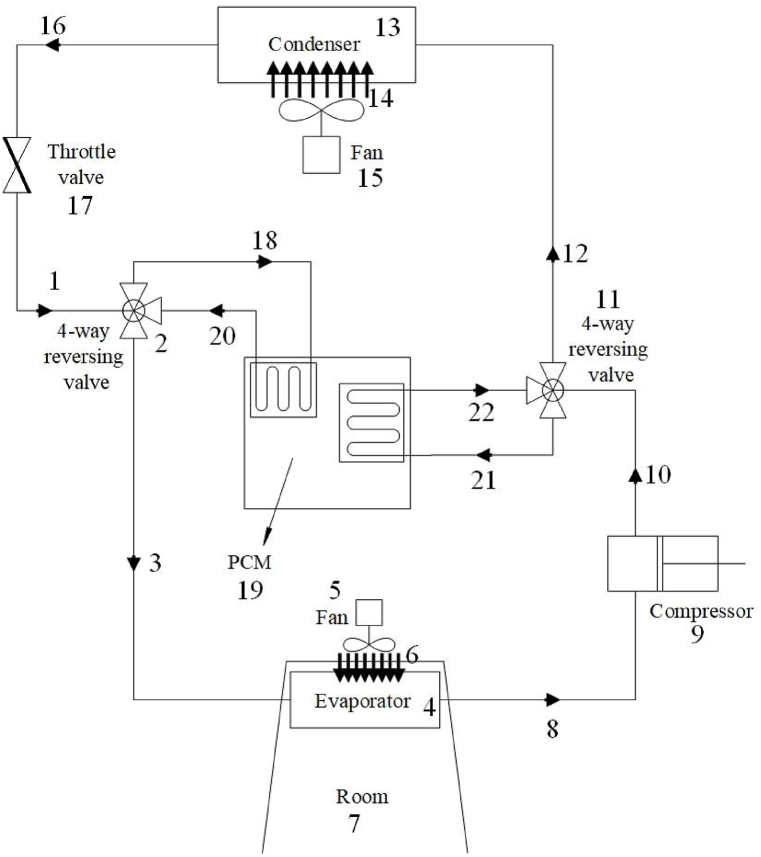
The schematic representation of the proposed system.

The proposed system, depicted in Fig. 1, operates in two distinct modes: a charging mode during off-peak hours and a discharging mode during on-peak hours. During periods of excess available cooling load energy, such as at night, the refrigerant stream leaving the throttle valve is directed to the PCM storage tank. Here, a portion of its cooling load energy is transferred to the PCM tank. Subsequently, the refrigerant stream enters the evaporator to meet the building's cooling load demand (charging mode). It then moves to the compressor and condenser to finalize the vapor compression cycle. During peak hours, if the building needs extra cooling energy, the stream exiting the compressor is initially directed to the PCM tank. This cools the refrigerant stream, leading to a reduction in condenser pressure and temperature. Consequently, the COP of the system improves, and its electrical consumption decreases.

As shown in Fig. 1, during off-peak hours, the refrigerant stream from the throttle valve (1) enters a four-way reversing valve (2). From there, it proceeds to the PCM storage tank (18), initiating the charging process by freezing the PCMs. Afterward, it continues to the evaporator (4), where the building is cooled by an adjacent air fan (5) positioned beside the evaporator heat exchanger. The outlet stream from the evaporator (8) is sent to the compressor (9), increasing the refrigerant's pressure and temperature. It subsequently flows into a second four-way reversing valve (11). In this mode, the refrigerant bypasses the PCM storage tank and is directed straight to the condenser (12), where it is cooled by an air fan that blows ambient air across the condenser heat exchanger. The refrigerant then enters the throttle valve (16). Operating the compressor at a lower pressure ratio during this phase results in reduced power consumption. This is aided by the PCM storage tank during on-peak hours. Using PCM lowers the temperature and pressure of the refrigerant stream at the outlet of the compressor. As a result, the condenser pressure is reduced during on-peak hours, requiring the compressor to use less energy to maintain the condenser pressure.

During on-peak hours, the refrigerant stream exiting the throttle valve (1) enters the four-way reversing valve (2), then proceeds to the evaporator (3) before reaching the compressor (8), where both pressure and temperature are increased. Subsequently, the compressor outlet stream enters the four-way reversing valve (11) and is then directed to the PCM storage tank (21). In this step, the refrigerant stream is cooled through heat exchange with the freezing PCM before moving on to the condenser (13). This results in a lower condenser pressure compared to conventional operation, ultimately reducing compressor energy consumption.

**Fig. 2:**
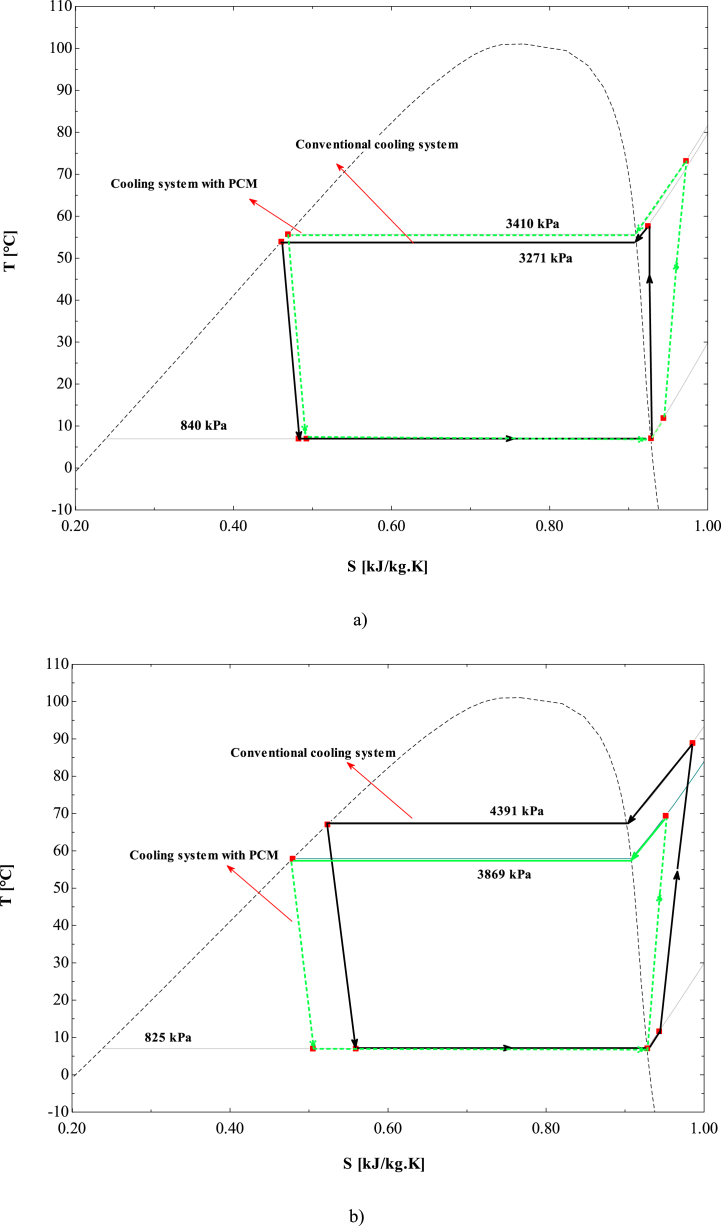
The T-S diagram of the proposed vapor compression cycle during a) charging mode and b) discharging mode.

The primary advantage of the proposed system lies in its ability to cool the compressor outlet stream during on-peak hours, resulting in an increase in the condenser's sub-cooled temperature and a decrease in condenser pressure. This effect contributes to an increase of both available cooling load capacity and the coefficient of performance (COP). The utilization of the PCM storage tank to cool the refrigerant stream entering the condenser results in decreased compressor power consumption. According to Fig. 2b, the reduction in condenser pressure during the discharging mode causes the T-S diagram to shift to lower values under two-phase conditions compared to the conventional system. This shift enhances the system's cooling capacity by increasing the sub-cooled temperature, which in turn raises the enthalpy value of the throttle valve outlet stream. Consequently, the desired system has the capacity to reduce peak loads and power consumption during on-peak hours when compared to a conventional vapor compression unit with the same required cooling load.

To clarify the process of incorporating the PCM storage tank into the vapor compression cycle for condenser cooling, the T-S diagram of the system during both charging (off-peak hours) and discharging (on-peak hours) phases at a random moment is presented in Fig. 2. The operation of the desired system during the charging mode is depicted in Fig. 2a. It is clear that the level of evaporator superheat is greater in the vapor compression system that incorporates the PCM tank. When the system is in charging mode and the PCM is freezing, heat is transferred to the refrigerant in the PCM storage tank, leading to a rise in the evaporator outlet temperature. As a result, the refrigerant enters the compressor at a higher temperature than it would in a conventional setup. This, in turn, increases the temperature of the compressor's outlet stream, which causes a corresponding rise in the condenser's saturation temperature and pressure.

The T-S diagram for the proposed system during the discharging mode is shown in Fig. 2b. By cooling the refrigerant stream prior to its entry into the condenser, both temperature and pressure within the condenser are significantly reduced. This cooling effect causes the refrigerant exiting the throttle valve to have a lower quality compared to that in a conventional system. Consequently, the temperature of the evaporator outlet stream decreases, which ultimately results in a lower temperature for the compressor outlet stream.

## 3 Experimental setup

**Fig. 3:**
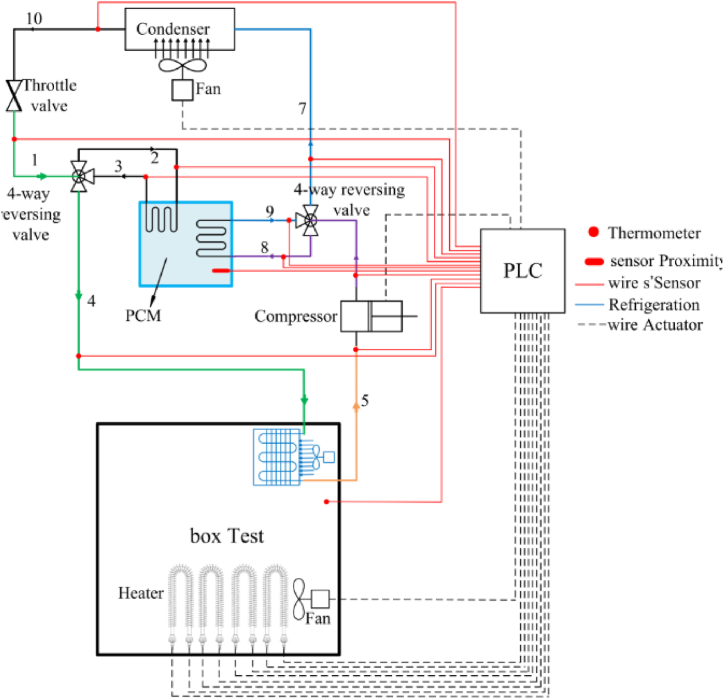
The process diagram of the vapor compression cycle integrated with a PCM storage tank (Equipment and data collection control system).

To evaluate the performance of the desired system, a test chamber is modeled and constructed to replicate the heat load conditions typically found inside buildings. Electrical heaters are utilized as consumers to mimic the required cooling load. Throughout the test, the vapor compression unit operated continuously, and precise control is maintained over the energy consumption of the electrical heaters to keep the test chamber temperature within the range of 20–22 °C. The experimental setup comprised a PCM storage tank, an air conditioning (AC) unit, a test chamber, electrical heaters, and a data collection system. The process diagram illustrating the configuration of the desired system is presented in Fig. 3.

### 3.1 Vapor compression system

A 12000 BTU air conditioning system has been used as a vapor compression system. The properties of the proposed AC unit are summarized in Table 1.

**Table 1:** The properties of the AC unit.

Parameter	Value	Parameter	Value
Cooling Capacity	12000 BTU	Gross Weight of Indoor Unit	11 kg
Heating Capacity	12400 BTU	Gross Weight of Outdoor Unit	28 kg
Cooling Rated Current	6.5 A	Bend of Indoor Evaporator	3
Heating Rated Current	6 A	Row of Indoor Evaporator	2
Maximum Power	1700 W	Pipe Diameter of Indoor Evaporator	7 mm
Maximum Current	8.6 A	Fan Motor Model of Indoor Unit	YYK18-4E
EER (Cooling)	8.82 BTU/(W.h)	Dimension of Indoor Unit	750 × 285 × 200 mm
COP (Heating)	10.33 BTU/(W.h)	Dimension of Indoor Packing	820 × 347 × 277 mm
Source of Power	220-240/50 HZ	Valve For Liquid	Dg4 mm
Refrigerant	R410A	Valve For Gas	Dg8 mm
Amount of Refrigerant	600 gr	Brand of Compressor	GMCC
Maximum Discharge Pressure	4.15 Mpa	Model of Compressor	ASM 130
Minimum Suction Pressure	1.15 Mpa	Fan Motor Model of Outdoor Unit	YDK25-6A
Fan Air Flow Rate	600 m^3^/h	Pipe Diameter of Condenser	7 mm
Noise of Indoor Unit	43 dB	Row of Condenser	1
Noise of Outdoor Unit	52 dB	Dimension of Outdoor Unit	650 × 500 × 240 mm

### 3.2 PCM storage unit

**Fig. 4:**
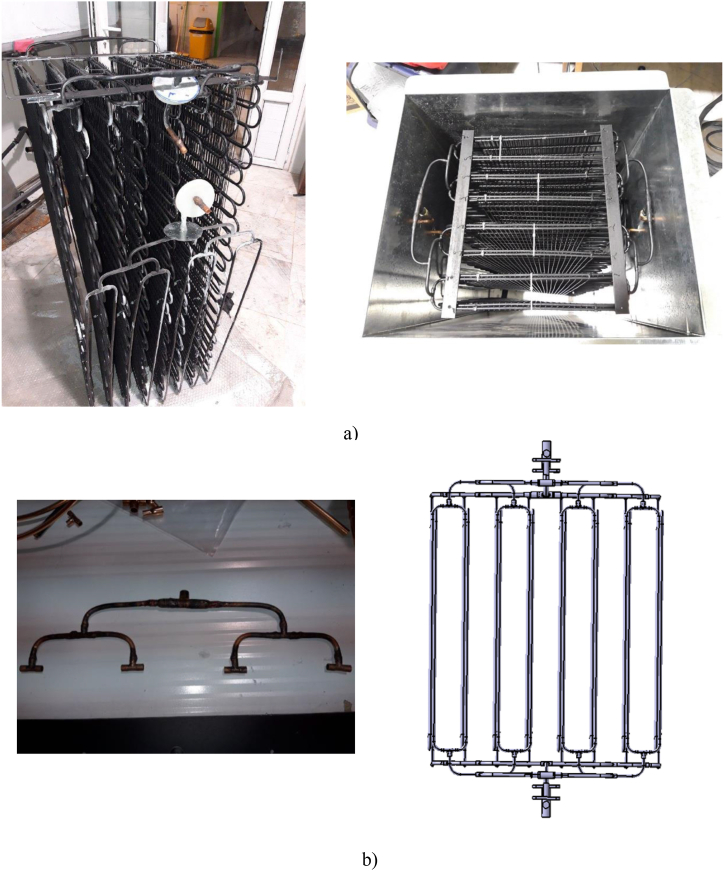
The PCM storage unit a) the tubes arrangement b) the heat exchanger headers.

The PCM storage unit is composed of a storage tank along with two tube heat exchangers. These heat exchangers are positioned side by side to create a counter-flow condition and are fully submerged in the PCM medium. The first heat exchanger is assigned to the charging process of the PCM medium, whereas the second one is used for the discharging process. The arrangement of the heat exchanger tubes is depicted in Fig. 4a. These heat exchangers consist of eight parallel tubes, with the refrigerant entering from the bottom of the tubes and exiting from the top during the charging process. Consequently, during charging, the lower section of the storage tank undergoes freezing first. The tubes are interconnected through the use of four headers, as illustrated in Fig. 4b. These headers are designed in such a way that pressure loss remains relatively consistent within each row. This ensures that the refrigerant flow remains uniform across each row. The storage tank is constructed as a rectangular cube, utilizing galvanized material, and sealed with tin metal. Additionally, a drainage valve has been incorporated into the storage tank. It's worth noting that the tubes are equipped with fins to enhance heat transfer within the PCM storage unit.

**Fig. 5:**
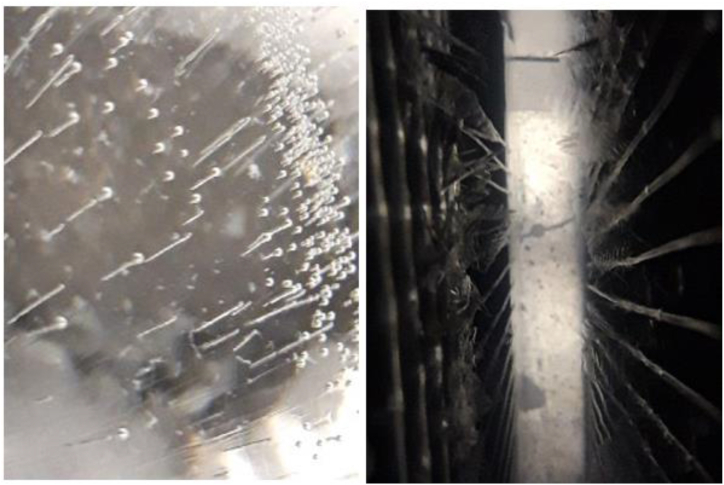
The effect of fins in ice formation on the tubes.

The ice formation on the tubes is illustrated in Fig. 5. As can be seen, the effect of fins heat transfer in the ice formation on the tubes is evident. The ice first formed along the fins.

Water has been used as the PCM in this experiment. The specifications of the PCM used in this study are provided in Table 2.

**Table 2:** The specification of the PCM.

Parameters	Value
Type of PCM	Water
Density (kg/m^3^)	1000
Melting Temperature (°C)	0
Latent Heat (kJ/kg)	333.7
Specific Heat (kJ/kg. K)	4.22
Amount of PCM (Lit)	300

### 3.3 Test chamber

For the construction of the test chamber, 5 cm thick polyurethane sandwich panels are employed. To ensure airtightness and prevent heat loss, silicone glue is applied to seal all edges of the test chamber. Additionally, a door is installed on one side of the test chamber, and rubber seals are utilized to create an effective seal. To replicate the thermal load conditions typically found in buildings, electrical heaters are installed. These electrical heaters are positioned in front of an air fan and are controlled by a dedicated controller. The controller regulates the ON/OFF cycles of the electrical heaters based on the system's cooling load requirements.

### 3.4 Controller system

Control and data collection for the desired system are managed by a Programmable Logic Controller (PLC), which oversees the operation of the compressor, electrical heaters, air fans, and solenoid valves. Additionally, the PLC system monitors and records data such as electrical heater power consumption, compressor power consumption, and temperature readings from various points in the system's cycle. The control system consists of PLC modules, including power, analog, and digital inputs and outputs, solid-state relays (SSR), a watt meter, ON/OFF switches, an emergency stop switch, a contactor, and current transformers (CT). All temperature sensors, pressure sensors, and capacitive proximity sensors are interfaced with the control system. Furthermore, all data can be monitored and controlled via a computer using the supervisory control and data acquisition (SCADA) system.

As previously mentioned, the control system is responsible for switching the system between charging and discharging modes based on both time and PCM temperature. Users have the flexibility to set the time intervals for charging and discharging. In this particular setup, the charging and discharging periods for the desired system are designated as 1:00 to 10:00 and 12:00 to 19:00, respectively, aligning with the electricity consumption patterns in Iran. During other times (19:00 to 1:00 and 10:00 to 12:00), the system operates in conventional mode. The transition between modes is facilitated by solenoid valves. If the cooling energy stored in the PCM storage tank is depleted during the discharging phase, the controller will automatically switch the system back to conventional mode.

The control system also maintains the temperature of the test chamber within the range of 20–22 °C by modulating the electrical heaters. It's important to note that the desired system consistently operates at its maximum available cooling load capacity to minimize the impact of system performance on thermal loads. If the test chamber temperature falls below 20° (indicating a high available cooling load), the controller will increase the electrical heater load. Conversely, if the test chamber temperature exceeds 22°, the controller will decrease the electrical heater load, thereby ensuring that the test chamber temperature remains within the specified range of 20–22 °C.

Furthermore, the control system manages the amount of ice formation within the PCM storage unit and controls the PCM storage temperature. During the charging mode (PCM freezing in the tank), the volume of PCM increases due to volumetric expansion, causing the liquid PCM level to rise. When the frozen PCM reaches approximately 95 % of the liquid PCM volume, a signal is sent to the control system via a capacitive sensor within the PCM storage tank. This signal prompts the control system to prevent further freezing, and the mode switches from charging to conventional mode.

### 3.5 Desired system experimental setup

**Fig. 6:**
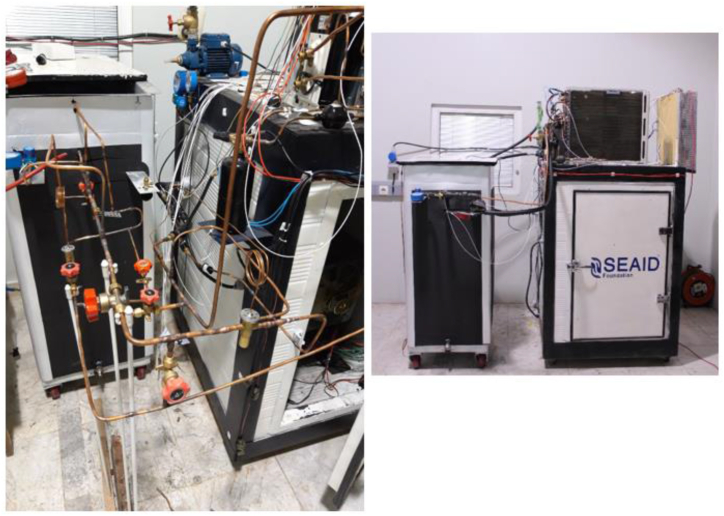
The desired system experimental setup.

The schematic diagram of the experimental setup for the proposed system is shown in Fig. 6. It's worth mentioning that within the condenser outlet stream refrigerant tube, a sight glass has been incorporated for monitoring purposes. Additionally, a three-way tube follows, which serves the dual function of pressure measurement and emergency refrigerant drainage. Subsequently, the refrigerant proceeds to pass through the filter dryer and the receiver.

## 4 Analytical modeling

This section provides a brief overview of the modeling, as the results of this study have been compared with the simulation outcomes from Ref. [27].

### 4.1 Heat exchangers

In this study, two types of heat exchangers are employed: an evaporator and a condenser. The modeling approach relies on the effectiveness-NTU (number of transfer units) method, which addresses both single-phase and two-phase conditions. Heat transfer rates (U-values) for these heat exchangers are calculated based on the standard geometries of typical evaporators and condensers. It is assumed that the outlet stream from the condenser is in a liquid state, and any pressure and heat losses within the heat exchanger are ignored. Table 3 provides the geometric specifications for the condenser and evaporator used in this analysis.

**Table 3:** The geometric properties of the heat exchangers utilized in this study.

Type of Heat Exchanger	Blow Rate of Fan (L/s)	Length of Tube (m)	Diameter of Tube (m)	Fin Number (#/in)
Condenser	1000	20	0.015	8
Evaporator	700	12	0.008	8

### 4.2 Compressor

This study models a rotary screw compressor that operates at 4000 rpm and has a volumetric inlet flow of 0.0495 L, consistent with modern vapor compression refrigeration systems. The model incorporates the isentropic, volumetric, and mechanical efficiencies of the compressor, while excluding any heat loss within the unit [30]. Details of the equations used for the compressor modeling can be found in Table 4.

**Table 4:** The equations used to model the compressor.

Parameter	Equation
Compressor Temperature Ratio	$\frac{T_{10}}{T_{9}}={\left(\frac{P_{cond}}{P_{evap}}\right)}^{\frac{\gamma -1}{\gamma }}$
The Refrigerant Mass Flow Rate	${\dot{m}}_{ref}=\eta_{v}\times \rho_{1}\times V_{G}\times \omega$
Volumetric Efficiency	$\eta_{v}=0.9207-0.0756\;\left(\frac{P_{10}}{P_{9}}\right)+0.0018{\left(\frac{P_{10}}{P_{9}}\right)}^{2}$
Compressor Power Consumption	${\dot{W}}_{comp}=\frac{{\dot{m}}_{ref}\left(h_{10,isen}-h_{9}\right)}{\eta_{total}}$
Compressor Total Efficiency	$\eta_{total}=\eta_{is}\eta_{mech}\eta_{elec}$
Compressor Isentropic Efficiency	$\eta_{is}=\begin{array}{c} 0.85-0.046667 \end{array}\;\left(\frac{P_{10}}{P_{9}}\right)$
Coefficient of Performance	$COP=\frac{{\dot{Q}}_{evap}}{{\dot{W}}_{comp}}$
Peak Load	${\dot{W}}_{maximum,comp}-\frac{\sum {\dot{W}}_{comp,conventional\;system}}{Operation\;Time}$
Peak Load Shaving	$\left(\;\frac{{Peak\;load}_{conventional\;system}-{Peak\;load}_{\;PCM\;integrated\;system}}{{Peak\;load}_{conventional\;system}}\right)\times 100$

### 4.3 PCM storage tank

This study investigates a PCM storage tank with a capacity of 300 L. The tank contains 20 horizontal pipes, each 1 m long. Refrigerant circulates through these pipes, facilitating heat transfer with the surrounding PCM. It is assumed that heat loss from the PCM storage tank to the surrounding environment is minimal. Additionally, the thermophysical properties of both the refrigerant and the PCM are treated as constant throughout the process. Geometric details of the PCM storage tank can be found in Table 5.

**Table 5:** The geometric specification of the PCM storage tank.

Item	Value
Tubes Number	20
Tubes Length (m)	1
Conductivity of Tube Wall (W/m. K)	111
Inner Diameter of Tube (m)	0.012
Outer Dimeter of Tube (m)	0.014

### 4.4 Software simulation

The dynamic modeling of the proposed system in this study was conducted using the Engineering Equation Solver (EES) software for a duration of 24 h, with a time step of 1 min. The charging phase, in which cold energy is stored in the PCM through solidification, is scheduled from 1:00 to 10:00. In contrast, the discharging phase, where cold energy is released from the PCM by melting, occurs between 12:00 and 19:00.

## 5 Results and discussions

This section evaluates the results of the experimental investigation of the proposed system. The electrical heater consumption (${\dot{Q}}_{evap}$), which denotes the amount of cooling load production by the desired system, and the compressor power consumption (${\dot{W}}_{comp}$), which is measured by the watt meter, are used to evaluate the system performance. The amount of COP is calculated from Equation (1).

(1) $$COP=\frac{{\dot{Q}}_{evap}}{{\dot{W}}_{comp}}$$

The experimental data is presented in the form of hourly averages. Initially, the conventional AC unit undergoes testing for a duration of 24 h. Subsequently, the AC unit, in combination with the PCM storage tank, is subjected to testing under identical operating conditions. Throughout these experiments, the temperature within the test chamber is precisely maintained within the range of 20–22 °C. This temperature regulation is achieved by controlling the electrical heaters, which effectively emulate the thermal load demands typically encountered in buildings and are managed by the controller. The PCM storage tank employed in the experiments has a volume of approximately 300 L and initially maintains a temperature of 25 °C. These experimental tests for both the conventional AC unit and the AC unit combined with the PCM storage tank were conducted over two consecutive days, during which the ambient temperatures were nearly identical. Charging and discharging of the PCM storage tank occurred within the time intervals of 1:00 to 10:00 and 12:00 to 19:00, respectively. Furthermore, the experimental results are juxtaposed with simulation data that employs a storage tank with water as the medium. This comparative analysis helps assess the performance of the PCM-based system in relation to a water-based system.

### 5.1 The conventional AC unit experimental results

**Fig. 7:**
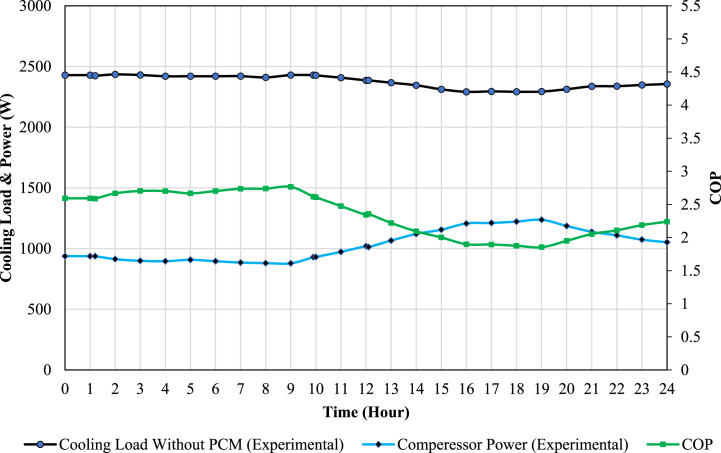
The experimental results of the available cooling load, compressor power consumption, and COP of the conventional AC unit.

The results pertaining to the conventional AC unit are visualized in Fig. 7. It's evident from the graph that during the middle of the day, corresponding to on-peak hours, certain trends are observed. Notably, the compressor power consumption experiences an increase, while the available cooling load production decreases. This behavior can be attributed to the rise in ambient temperature that typically occurs during on-peak hours. Consequently, the condenser's pressure and temperature also increase. Consequently, the Coefficient of Performance (COP) shows a significant decrease during these specific hours.

### 5.2 The AC unit combined with PCM storage experimental results

**Fig. 8:**
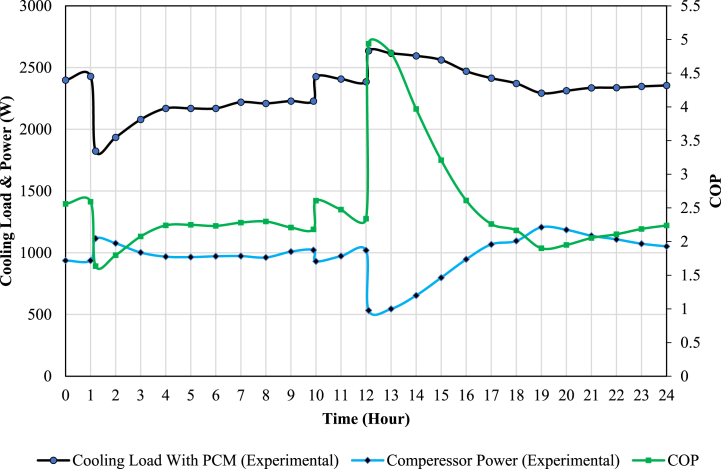
The experimental results of the available cooling load, compressor power consumption, and COP of the AC unit combined with the PCM storage tank.

Fig. 8 displays the experimental results for the air conditioning unit integrated with the PCM storage tank. In this configuration, a portion of the cooling load production is stored in the PCM storage tank during the charging process, which results in a reduction in the available cooling load during the specified time interval (1:00 to 10:00). Consequently, there is an increase in compressor power consumption during this phase. This increase can be attributed to the rise in the evaporator outlet's superheated refrigerant temperature, which has an impact on compressor power consumption.

Conversely, during the discharging mode (12:00 to 19:00), the compressor power consumption decreases while the available cooling load production increases. This phenomenon is attributed to the condenser's cooling effect by the PCM storage tank, which in turn reduces both the condenser temperature and pressure.

### 5.3 Temperature and pressure variations in different cycle spots

**Fig. 9:**
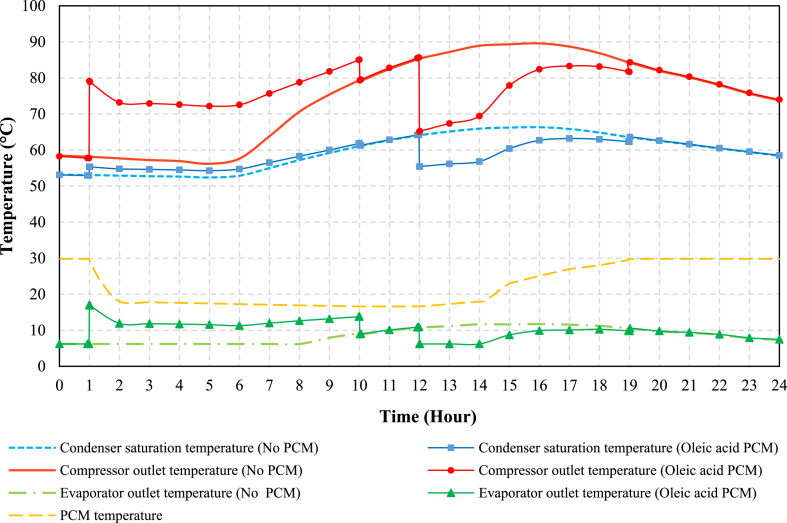
The simulation results of various spots of the cycle for oleic acid PCM and conventional modes.

The temperatures of the condenser, evaporator, and compressor outlet in both PCM and conventional modes are depicted in Fig. 9. Significantly, during the charging process in PCM mode, the saturation outlet temperatures of the condenser and compressor are found to be higher than those in conventional mode. This increase can be attributed to the enhanced heat transfer rate from the PCM to the refrigerant during the charging process, resulting in a greater degree of superheat in the evaporator outlet stream. In simpler terms, more thermal load is transferred to the refrigerant, causing an increase in the evaporator outlet temperature, which subsequently leads to higher compressor outlet temperatures. Consequently, this has an effect on the condenser's saturation temperature.

Conversely, during the discharging process, both the saturation compressor and condenser outlet temperatures experience a reduction. This is due to the fact that the condenser is more effectively cooled during discharging, leading to a decrease in condenser pressure. As a result, both the compressor outlet temperature and the condenser's saturation temperature decrease during this phase.

**Fig. 10:**
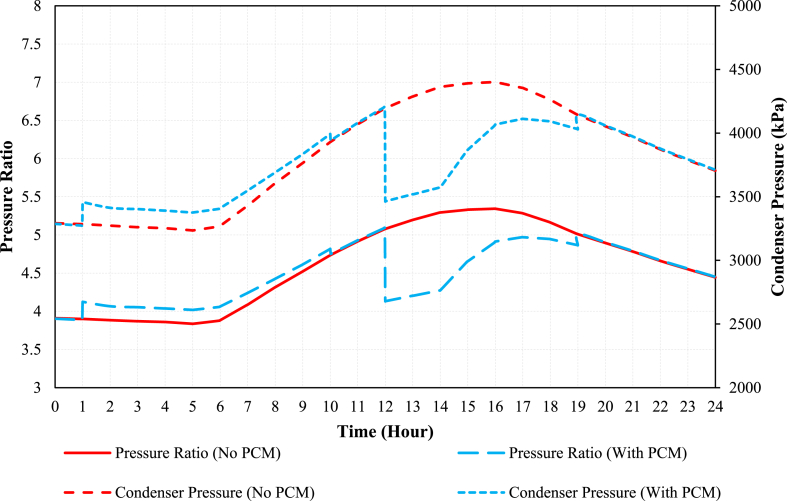
The comparison of pressure ratios in PCM and conventional modes.

The pressure ratios, specifically the ratio of condenser pressure to evaporator pressure as well as the condenser absolutes pressure, are presented in Fig. 10 for both PCM and conventional modes. Observations from the graph indicate that the pressure ratio experiences an increase during the charging process, resulting in a decrease in the Coefficient of Performance (COP). This behavior is directly linked to the rising condenser pressure, as mentioned earlier. In contrast, during the discharging process in PCM mode, the pressure ratio undergoes a significant reduction. This reduction can be attributed to the decreasing condenser pressure, as previously discussed.

### 5.4 Comparing simulation and experimental results

**Fig. 11:**
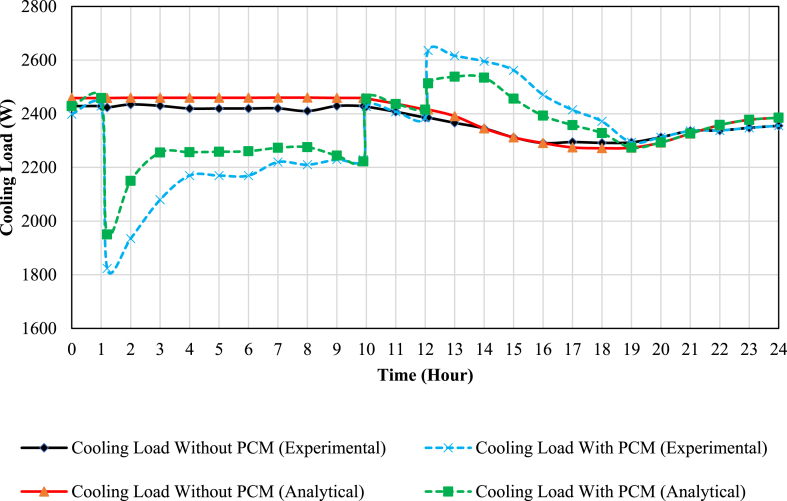
The comparison of simulation and experimental results of the AC unit available cooling load in PCM and conventional modes.

**Fig. 12:**
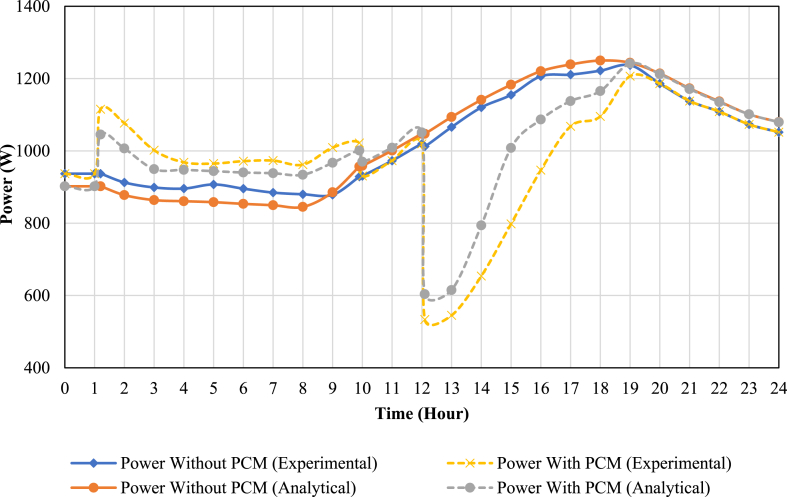
The comparison of simulation and experimental results of the AC unit compressor power consumption in PCM and conventional modes.

Fig. 11, Fig. 12 show the available cooling load and power consumption of the air conditioning unit in both PCM and conventional modes, respectively. These experimental values are then compared with the simulation data from a previous study [27]. The graphs clearly demonstrate a strong correlation between the simulation and experimental data. The comprehensive results are further summarized in Table 6, Table 7. These tables provide details on the Coefficient of Performance (COP), available cooling load, and compressor power consumption during both the charging and discharging processes. Additionally, daily values for these parameters are presented, offering a comprehensive overview of the system's performance characteristics.

**Table 6:** The amount of COP, compressor power consumption, and available cooling load for conventional AC unit based on experimental and simulation data.

Type of Data	Time	Compressor Energy Consumption (kWh)	Available Cooling Load (kWh)	COP
Experimental Data	Daily	26.9	58.4	2.17
	Charging (1–10)	9.1	24.4	2.68
	Discharging (12–19)	7.9	16.3	2.06
Simulation Data	Daily	25.8	59.8	2.31
	Charging (1–10)	8.2	25.6	3.12
	Discharging (12–19)	8.3	16.5	1.98
Error (%)		4	2.3	6.4
		9.8	1.2	16.4
		5	1.2	3.8

**Table 7:** The amount of COP, compressor power consumption, and available cooling load for AC unit combined with PCM tank based on experimental and simulation data.

Type of Data	Time	Compressor Energy Consumption (kWh)	Available Cooling Load (kWh)	COP
Experimental Data	Daily	24.1	56.2	2.33
	Charging (1–10)	10.6	20.5	1.93
	Discharging (12–19)	5.5	18.9	3.4
Simulation Data	Daily	25.2	57.3	2.27
	Charging (1–10)	9.8	21.2	2.1
	Discharging (12–19)	6.4	17.3	2.7
Error (%)		4	1.9	2.5
		7.5	3.4	8.8
		16.3	8.4	26

As observed, incorporating a PCM storage tank into the conventional AC unit improves the daily coefficient of performance (COP) by approximately 7 % (from 2.17 to 2.33). It should be noted that using the PCM storage tank decreases the daily available cooling load by about 3.7 % (from 58.4 to 56.2 kWh). Moreover, the daily compressor energy consumption reduces by about 10.3 % (from 26.9 to 24.1 kWh).

There are errors between simulation and experimental data, which their reasons might be as follows:

• The evaporator temperature was considered −5° in the simulation study, while it is not constant in the experimental study due to the fluctuation in the real system.
• Since the operating compressor conditions and its characteristic curve were not available for the simulation study, the analytical formulas presented in Ref. [27] may be slightly different from the actual conditions of compressor operation.
• The test chamber heat loss cannot be calculated, which may affect the results.
• The PCM storage tank was considered adiabatic in the simulation study, while it has heat loss in the experimental study.
• The measurement equipment may have errors.
• The simplification assumptions in the simulation study.
• Different systems operating conditions (for instance, the different amounts of cycle refrigerant flow in simulation and experimental studies).

## 6 Conclusion

This study evaluates a vapor compression refrigeration cycle that incorporates a Phase Change Material (PCM) storage tank. The primary purpose of the PCM storage tank is to manage peak cooling loads effectively. During off-peak hours, when cooling demand is low, part of the cooling capacity produced by the system is used to charge the PCM storage tank. The thermal energy stored in the PCM is then utilized during on-peak hours, when the cooling demand reaches its peak. This approach resulted in an enhancement of both the Coefficient of Performance (COP) and the available cooling capacity during on-peak hours. The evaluation of the system is carried out in two distinct modes: PCM mode and conventional mode, spanning a 24-h period. Initially, a 24-h test of the conventional air conditioning unit is conducted. Subsequently, the impact of introducing PCM technology to the conventional AC unit under identical conditions is assessed. To emulate a building environment, a test chamber is designed to maintain a consistent temperature of approximately 20 °C. This temperature control is achieved through the operation of electrical heaters, which were controlled by a dedicated controller. The PCM storage tank had a capacity of around 300 L and an initial temperature of 25 °C. Water was chosen as the PCM medium. The experimental tests for both the conventional AC unit and the AC unit incorporating the PCM storage tank are conducted over two consecutive days, during which the ambient temperature remained consistent. Charging and discharging of the PCM storage tank are scheduled between 1:00 to 10:00 and 12:00 to 19:00, respectively. Additionally, a comparison is made between the experimental results and simulation data. The primary findings of the experimental evaluation are as follows:

➢ Incorporating the PCM storage tank into the conventional AC unit yielded a notable enhancement in daily COP, increasing it by approximately 7 % (from 2.17 to 2.33). Furthermore, it resulted in a reduction in daily available cooling capacity and compressor energy consumption by roughly 3.7 % (from 58.4 to 56.2 kWh) and 10.3 % (from 26.9 to 24.1 kWh), respectively.
➢ The experimental results of the conventional AC unit closely aligned with the simulation outcomes, with percentage errors of 6.4 % for daily available cooling capacity, 2.3 % for compressor energy consumption, and 4 % for COP.
➢ Similarly, the experimental results of the AC unit combined with the PCM storage tank exhibited strong agreement with the simulation results, featuring percentage errors of 2.5 % for daily available cooling capacity, 1.9 % for compressor energy consumption, and 4 % for COP.

## CRediT authorship contribution statement

**Alireza Riahi:** Writing – original draft, Methodology, Investigation. **Mohammad Behshad Shafii:** Supervision, Conceptualization.

## Data availability statement

The data obtained from this study are incorporated within the manuscript details.

## Declaration of competing interest

The authors declare that they have no known competing financial interests or personal relationships that could have appeared to influence the work reported in this paper.

## Figures and Tables

**Table:** 

**Abbreviation**	
AC	Air Conditioning
COP	Coefficient of Performance
CT	Current Transformers
EES	Engineer Equation Solver
IEA	International Energy Agency
NTU	Number of Transfer Unit
PCM	Phase Change Material
PLC	Programmable Logic Controller
SCADA	Supervisory Control and Data Acquisition
SSR	solid-state relays
**Symbols**	
h	Enthalpy (kJ/kg. K)
$\dot{m}$	Mass Flow Rate (kg/s)
P	Pressure (kPa)
${\dot{Q}}_{evap}$	Evaporator Cooling Load (kW)
T	Temperature (°C)
$V_{G}$	Compressor Inlet Volume (m^3^)
${\dot{W}}_{comp}$	Compressor Power Consumption (kW)
**Subscript**	
9	Compressor Inlet
10	Compressor Outlet
C	Cold fluid flow
Comp	Compressor
cond	Condenser
elec	Electrical
Evap	Evaporator
in, i	Inlet
is, isen	Isentropic
mech	Mechanical
o, out	Outlet
**Greek Letter**	
$\eta_{is}$	Isentropic efficiency
$\eta_{v}$	Volumetric efficiency
ρ	Density (kg/m^3^)
ω	Compressor speed (rpm)
